# The Alleged Discovery of the Cause of Tuberculosis

**Published:** 1882-06

**Authors:** 


The Alleged Discovery of the Cause of Tuberculosis.—The recent discoveries of Dr. Koch, of Berlin, of which we give an abstract in another part of this issue, deserve the most thoughtful consideration. The wide-spread prevalence and the excessive fatality of tuberculosis must, of necessity, invest all attempts to search out its cause with the greatest interest. It is, therefore, with much satisfaction that we give place to Professor Tyndall's enthusiastic letter, although we cannot share his enthusiasm. In spite of Professor Tyndall and Dr. Koch, we cannot yet agree that the germ-theory has been demonstrated. Even the bacterial pathogeny of splenic and relapsing fevers has received some severe back-sets recently from Prof. Fokker and Dr. Carter. If we add to this the fact that Dr. Koch, Prof. Baumgarten, and Prof. Aufrecht, who all claim priority in the discovery of the bacillus tuberculosis, are not agreed upon the nature, shape, and size of the parasite, although admitting its etiological relation to the disease, we still find abundant reason for declining to accept Dr. Koch's decision as final.

